# Rapidly enlarging facial tumors

**DOI:** 10.1016/j.jdcr.2025.05.025

**Published:** 2025-06-18

**Authors:** Jeffrey Weiner, Jeffrey Meyer, Jong Bin Park, Cole Sterling, Sima Rozati

**Affiliations:** Department of Dermatology, Johns Hopkins University School of Medicine, Baltimore, Maryland

**Keywords:** cutaneous T-cell lymphoma, facial tumors, mycosis fungoides, radiation therapy

## Case description

**Fig 1:**
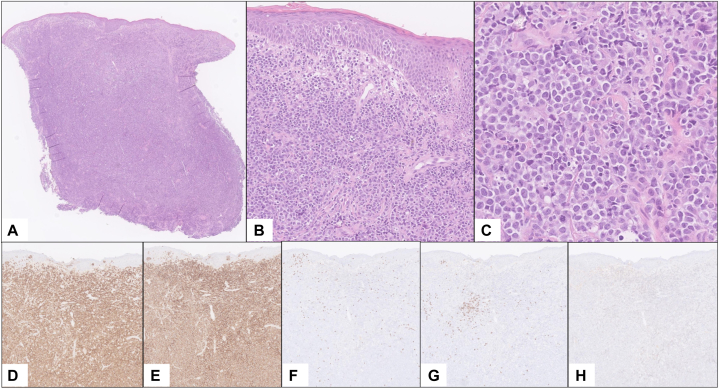
Histopathology of facial tumor biopsy. **A,** Low, **(B)** medium, and **(C)** high-power H&E staining demonstrate an epidermotropic lymphoid infiltrate with large, atypical cells exhibiting irregular hyperconvoluted nuclei, vesicular chromatin, and prominent nucleoli comprising > 25% of the infiltrate. Immunohistochemistry staining reveals the infiltrate to be **(D)** CD3+, **(E)** CD4+, **(F)** CD8−, **(G)** CD20−, and **(H)** CD30−, confirming the diagnosis of mycosis fungoides with large-cell transformation. *CD*, Cluster of Differentiation; *H&E*, hematoxylin and eosin.

**Fig 2:**
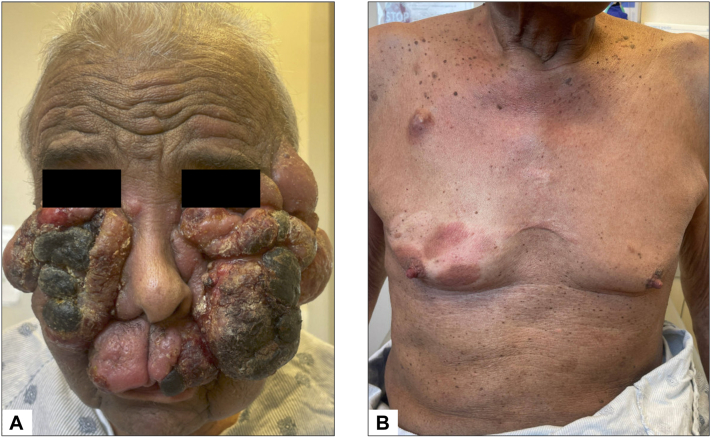
Clinical presentation prior to treatment with radiotherapy and romidepsin. **A,** The face exhibits multiple violaceous tumors with hemorrhagic and *grayish* crusting over the bilateral malar cheeks and temples. **B,** The trunk displays erythematous patches and erythematous-to-violaceous plaques.

An 80-year-old man presented with a 4-month history of rapidly enlarging facial tumors. Examination revealed multiple violaceous tumors with hemorrhagic and greyish crusting involving the bilateral malar cheeks/temples. The trunk and extremities were covered with diffuse erythematous patches and erythematous-to-violaceous plaques, some with overlying scale that had been present for years. He had 1 to 2 cm cervical, axillary, and inguinal lymphadenopathy but denied B-symptoms. A biopsy of a facial tumor revealed an epidermotropic Cluster of Differentiation (CD) 3+CD4+CD8-CD20-CD30- lymphoid infiltrate, with large lymphocytes exhibiting irregular hyperconvoluted nuclei, vesicular chromatin, and prominent nucleoli composing > 25% of the infiltrate (Fig 1). This confirmed the diagnosis of mycosis fungoides with large-cell transformation. Staging workup with positron emission tomography/computed tomography revealed increased fluorodeoxyglucose uptake in the axillary and inguinal lymph nodes and peripheral blood flow cytometry showed no immunophenotypic abnormalities, corresponding to mycosis fungoides stage IIB (tumor stage 3, nodes unknown, no metastasis to viscera, blood stage 0). The patient had recently completed treatment with brentuximab vedotin resulting in a partial response followed by disease progression. He was transitioned to mogamulizumab by his local oncologist but experienced rapid worsening of his tumors (Fig 2).

**Question: Of the following options, which is the preferred next treatment for this patient?**

**A.** Localized radiotherapy ± single-agent systemic treatment
**B.** Cyclophosphamide, hydroxydaunorubicin (doxobicin), oncovin (vincristine), and prednisone
**C.** Narrowband ultraviolet B or Psoralen + ultraviolet A phototherapy
**D.** Extracorporeal photopheresis
**E.** Topical mechlorethamine

## Discussion

Mycosis fungoides presents as patches, plaques, or tumors with epidermotropic T-cell infiltrates. Large, atypical lymphocytes making up more than 25% of the infiltrate in a biopsy constitutes large-cell transformation of mycosis fungoides, which may be highlighted by CD30 expression, with CD30 negativity portending a worse prognosis. This variant can have a more aggressive and refractory course, including the rapidly enlarging tumors seen in this patient.^1^

Patients with mycosis fungoides should undergo staging to guide prognosis and treatment by assessing the possible compartments of disease: skin, lymph nodes, visceral organs, and blood. Positron emission tomography/computed tomography is the standard imaging modality for identifying lymph node and visceral organ involvement, and peripheral blood flow cytometry is used for assessing blood involvement.^2^

**Fig 3:**
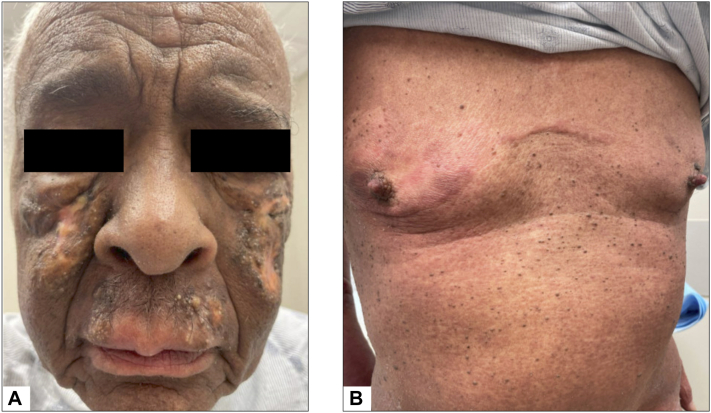
Clinical response 2 months after treatment with radiotherapy and romidepsin. **A,** The face shows near-complete resolution of tumors. **B,** The trunk demonstrates mainly hyperpigmented patches and partial resolution of plaques.

For this patient, Choice A (localized radiotherapy ± single-agent systemic treatment) is the preferred next treatment. Low-dose localized radiotherapy provides significant palliative benefits for tumor-stage mycosis fungoides, particularly when tumors impair activities of daily living. Radiation prescriptions such as 7 to 8 Gy in a single fraction or 4 Gy × 2 fractions are associated with high response rates and effective palliation.^3^^,^^4^ Systemic treatment is appropriate for widespread patch/plaque disease, nodal or visceral involvement, rapidly progressive disease, or refractory cases.^5^ Because our patient’s facial tumors compromised his sight and balance, we prioritized symptom control with localized photon beam radiotherapy (4 Gy × 3 fractions), resulting in a local complete response (Fig 3, *A*). However, due to worsening lymphadenopathy and persistent patches/plaques on the trunk and extremities, systemic treatment with romidepsin was initiated, leading to partial resolution of the plaques on the trunk (Fig 3, *B*).

Multiagent chemotherapy is not the preferred treatment due to its limited efficacy for tumors, short duration of response, and higher toxicity compared to targeted therapies.^5^ Phototherapy and topical mechlorethamine, while effective for patch/plaque-stage mycosis fungoides, lack the penetration required for tumors. Extracorporeal photopheresis targets mainly malignant T-cells in the blood and can take a few months to achieve response, making it much less effective as monotherapy for this patient’s progressive disease without blood involvement.

## Conflicts of interest

None disclosed.
